# Potential Interactions of Calcium-Sensitive Reagents with Zinc Ion in Different Cultured Cells

**DOI:** 10.1371/journal.pone.0127421

**Published:** 2015-05-26

**Authors:** Koichi Fujikawa, Ryo Fukumori, Saki Nakamura, Takaya Kutsukake, Takeshi Takarada, Yukio Yoneda

**Affiliations:** Laboratory of Molecular Pharmacology, Division of Pharmaceutical Sciences, Kanazawa University Graduate School of Medical, Pharmaceutical and Health Sciences, Kanazawa, Ishikawa 920–1192, Japan; University of Debrecen, HUNGARY

## Abstract

**Background:**

Several chemicals have been widely used to evaluate the involvement of free Ca^2+^ in mechanisms underlying a variety of biological responses for decades. Here, we report high reactivity to zinc of well-known Ca^2+^-sensitive reagents in diverse cultured cells.

**Methodology/Principal Findings:**

In rat astrocytic C6 glioma cells loaded with the fluorescent Ca^2+^ dye Fluo-3, the addition of ZnCl_2_ gradually increased the fluorescence intensity in a manner sensitive to the Ca^2+^ chelator EGTA irrespective of added CaCl_2_. The addition of the Ca^2+^ ionophore A23187 drastically increased Fluo-3 fluorescence in the absence of ZnCl_2_, while the addition of the Zn^2+^ ionophore pyrithione rapidly and additionally increased the fluorescence in the presence of ZnCl_2_, but not in its absence. In cells loaded with the zinc dye FluoZin-3 along with Fluo-3, a similarly gradual increase was seen in the fluorescence of Fluo-3, but not of FluoZin-3, in the presence of both CaCl_2_ and ZnCl_2_. Further addition of pyrithione drastically increased the fluorescence intensity of both dyes, while the addition of the Zn^2+^ chelator N,N,N',N'-tetrakis(2-pyridylmethyl)ethane-1,2-diamine (TPEN) rapidly and drastically decreased FluoZin-3 fluorescence. In cells loaded with FluoZin-3 alone, the addition of ZnCl_2_ induced a gradual increase in the fluorescence in a fashion independent of added CaCl_2_ but sensitive to EGTA. Significant inhibition was found in the vitality to reduce 3-(4,5-dimethyl-2-thiazolyl)-2,5-diphenyl-2H-tetrazolium bromide in a manner sensitive to TPEN, EDTA and BAPTA in C6 glioma cells exposed to ZnCl_2_, with pyrithione accelerating the inhibition. Similar inhibition occurred in an EGTA-sensitive fashion after brief exposure to ZnCl_2_ in pluripotent P19 cells, neuronal Neuro2A cells and microglial BV2 cells, which all expressed mRNA for particular zinc transporters.

**Conclusions/Significance:**

Taken together, comprehensive analysis is absolutely required for the demonstration of a variety of physiological and pathological responses mediated by Ca^2+^ in diverse cells enriched of Zn^2+^.

## Introduction

A prevailing view is that the excitatory amino acid neurotransmitter L-glutamic acid (Glu) plays a crucial role in neuronal development [1], neuronal plasticity [2] and neuronal cytotoxicity [3,4] through a mechanism relevant to the incorporation of extracellular Ca^2+^ across cell membranes [5,6] after activation of particular ionotropic receptor subtypes, such as N-methyl-D-aspartate receptor (NMDAR), in the mammalian brain. A large number of probes and reagents have been developed for the purpose to confirm and to validate the possible involvement of intracellular free Ca^2+^ in a variety of biological phenomena associated with activation of different transmembrane receptors for extracellular signals. For example, Calcium Green-1, Fura-2, Fluo-3, Fura-6F and others have been used to detect free Ca^2+^ levels in different cells exposed to a variety of extracellular stimuli *in vitro* [7,8]. An acetoxymethyl (AM) ester of rhodamine-2 (Rhod-2) is able to easily penetrate cellular membranes for the intracellular cleavage of AM ester and subsequent oxidization to Rhod-2 for Ca^2+^-dependent fluorescence in mitochondrial environments [9,10].

In addition to these fluorescent indicators useful for detecting free Ca^2+^ levels in different subcellular locations, a membrane permeable AM ester of 1,2-bis(o-aminophenoxy)ethane-N,N,N',N'-tetraacetic acid (BAPTA) has been used to chelate free Ca^2+^ in the cytoplasm with both membrane-impermeable EDTA and EGTA being a chelator for extracellular free Ca^2+^ [11]. In contrast, 5-(methylamino)-2-[[(2S,3R,5R,8S,9S)-3,5,9-trimethyl-2-[1-oxo-1-(1H-pyrrol-2-yl)propan-2-yl]-1,7-dioxaspiro[5.5]undecan-8-yl]methyl]-1,3-benzoxazole-4-carboxylic acid (A23187) is believed to create a complex with divalent cations as an ionophore required for the selective entry of extracellular free Ca^2+^ in diverse cell membranes [12,13].

However, recent studies have shown the potential interaction of the aforementioned fluorescent Ca^2+^ indicators with other free divalent cations such as Zn^2+^ in different situations [7,8]. Although free Zn^2+^ is released from a variety of Zn^2+^-binding proteins essential for the maintenance of diverse cellular functions and integrities in response to oxidative stress [14–16], emerging evidence is now accumulating for the physiological and pathological significance of Zn^2+^ in homeostatic functional modulations of the brain. In murine hippocampal slices, Zn^2+^ is released together with Glu into synaptic clefts in a Ca^2+^-dependent manner upon stimulation of Schaffer collateral fibers [17]. Activation of ionotropic Glu receptors leads to increased intracellular free Zn^2+^ levels with high toxicity via channels and transporters for Ca^2+^ in neurons cultured in the presence of Zn^2+^ [18–20]. Extracellular Zn^2+^ is shown to directly and progressively permeate NMDAR channels permeable for Ca^2+^ [21], in addition to inhibiting the opening of the channels [22,23] through an action site at a particular NMDAR subunit [24]. Moreover, Zn^2+^ is supposed to play a critical role in the pathogenesis of different neurodegenerative diseases such as Alzheimer’s disease [25] and amyotrophic lateral sclerosis (ALS) [26]. Upregulation of the Ca^2+^/Zn^2+^ binding protein S100A6 is similarly seen in astrocytes of autopsied brains from patients with Alzheimer’s disease and ALS [27].

These previous findings prompted us to investigate the specificity of several reagents used for confirming and validating the essential requirement for free Ca^2+^ in different biological systems for decades, in terms of intracellular mobilization and cellular survival using a variety of cloned lines of cells found in the brain.

## Materials and Methods

### Materials

Rat astrocytic C6 glioma cells and human embryonic kidney (HEK)-293 cells were purchased from RIKEN Cell Bank (Saitama, Japan). Mouse embryonal carcinoma P19 cells were obtained from ATCC (Manassas, VA, USA). Mouse microglial BV-2 cells are a generous gift from Dr. Eui-Ju Choi (Korea University, Seoul, Korea) [28]. Poly-L-lysine, all-*trans* retinoic acid (ATRA), Hoechst33342, propidium iodide (PI), A23187, 3-[4,5-dimethylthiazol-2-yl]-2,5-diphenyltetrazolium bromide (MTT), 2-mercaptopyridine N-oxide sodium (pyrithione) and N,N,N’,N’-tetrakis(2-pyridylmethyl)ethylenediamine (TPEN) were purchased from Sigma-Aldrich fine Chemicals (St. Louis, MO, USA). Acetoxymethyl esters of Fluo-3, Rhod-2 and FluoZin-3 were provided by Molecular Probes (Eugene, OR, USA). Both EGTA and BAPTA-AM were supplied by Dojindo (Kumamoto, Japan). Dulbecco’s Modified Eagle Medium (DMEM) and alpha minimal essential medium (αMEM) were provided by Wako (Osaka, Japan). EDTA was purchased from Nacalai Tesque (Kyoto, Japan). Other chemicals used were all of the highest purity commercially available.

### Cell culture

Rat astrocytic C6 glioma cells [29], mouse neuroblastoma Neuro2A cells [30] and mouse microglial BV-2 cells [31] were cultured in DMEM supplemented with a 10% fetal bovine serum (FBS) as described elsewhere. Neuro2A cells were subjected to medium change with DMEM supplemented with 2% FBS and 20 μM ATRA for commitment to the neuronal lineage. Mouse embryonal carcinoma P19 cells [32] were cultured in αMEM supplemented with FBS, followed by further culture in the presence of 0.5 μM ATRA for 4 days to promote commitment to the neural lineage under floating conditions and subsequent trypsinization for dispersion. Cultures were always maintained in a humidified atmosphere of 5% CO_2_ and 95% air at 37°C.

### Orchestration of acquired NMDAR channels

In addition to several cell lines described above, we used rat NMDAR subunits cloned into expression vectors to artificially orchestrate membrane receptor channels highly permeable for Ca^2+^ [33]. The plasmid constructs pcDNAI-GluN2A and pcDNA3.1-GluN1-1a were generous gifts from Dr. Jon W. Johnson (Department of Neuroscience, University of Pittsburgh, PA, USA) [34]. HEK293 cells were grown in DMEM supplemented with 5% FBS before transfection as described previously [35]. Cells were transfected at a 1:3 ratio with GluN1-1a and GluN2A subunit expression vectors by the calcium phosphate method in DMEM with 5% FBS, followed by rinsing with recording medium containing 129 mM NaCl, 4 mM KCl, 1 mM MgCl_2_, 2 mM CaCl_2_, 4.2 mM glucose and 10 mM HEPES (pH 7.4).

### Fluorescence intensity and imaging

Medium was changed with recording medium once more, followed by incubation with 3 μM Fluo-3 AM for determination of intracellular free Ca^2+^ levels [33] and/or 3 μM Rhod-2 AM for determination of mitochondrial free Ca^2+^ levels [10] along with 30 nM Pluronic F-127 and subsequent washing twice for monitoring the individual fluorescence with an interval of 1 min using a confocal laser-scanning microscope (LSM510, Carl Zeiss, Jena, Germany). Fluorescence intensity was normalized after the addition of the Ca^2+^ ionophore A23187 at 10 μM at the end of each experiment for subsequent quantitative analysis. Fluorescence images with Fluo-3 and Rhod-2 were collected using excitation wavelengths of 488 nm and 543 nm, respectively.

Similarly, cells were loaded with 30 nM Pluronic F-127 and 3 μM FluoZin-3 AM for monitoring the fluorescence intensity using a confocal laser-scanning microscope. The calcium ionophore A23187 was then added at 10 μM to obtain the maximal fluorescence for quantitative normalization. Fluorescence images with FluoZin-3 were collected using an excitation wavelength of 488 nm. The parameters of illumination and detection were digitally controlled to keep the same settings throughout the experiments. For quantitative analysis of A23187 fluorescence, images were invariably quantified using ImageJ software (NIH, Bethesda, MD, USA) as the mean gray value in a visual filed selected at random 3 min after the addition of 10 μM A23187. Excitation and emission wavelengths for each fluorescent probe are as follows: Fluo-3, excitation = 505 nm, emission = 526 nm; Fluo-Zin-3, excitation = 494 nm, emission = 516 nm; Rhod-2, excitation = 552 nm, emission = 578 nm.

### Determination of cell viability

Cells were usually exposed to ZnCl_2_ at different concentrations in the presence of a variety of chemicals for 60 min, followed by further culture for an additional 24 h and subsequent determination of cell viability with MTT reduction assays unless otherwise indicated [33]. In brief, culture medium was replaced with phosphate-buffered saline containing 0.5 mg/ml MTT and incubated for 1 h at 37°C. Cells were then solubilized in a lysis solution containing 99.5% isopropanol and 0.04 M HCl. The amount of MTT formazan product was determined by measuring the absorbance at 550 nm on a microplate reader. Relative values were calculated by percentages over control values obtained in a parallel control experimental group. Cells were also exposed to ZnCl_2_ for different periods of 10 to 60 min, followed by further culture for an additional period of 0.5 to 24 h for subsequent MTT reduction assays as needed.

Cell viability was also examined by double staining with the membrane-permeable fluorescent dye Hoechst33342 at 10 μg/ml and the membrane-impermeable dye PI at 5 μg/ml for DNA. Cultured cells were washed by culture medium and incubated with both dyes in culture medium for 10 min. Cells were then observed using an epifluorescent microscope (BZ-8100; Keyence, Osaka, Japan). The numbers of cells stained with Hoechst33342 and PI were individually counted in five different visual fields chosen at random per each well, to calculate percentages of PI-positive cells over the total cells stained with Hoechst33342 as an index of dead cells [10].

### Real-time based quantitative polymerase chain reaction (qPCR)

Total RNA was extracted from cells, followed by synthesis of cDNA with reverse transcriptase and oligo dT primer. The cDNA samples were then used as templates for real-time qPCR analysis, which was performed on an MX3005P instrument (Agilent Technologies, Santa Clara, CA, USA), by using specific primers for each gene as summarized in Table 1. Expression levels of the genes examined were normalized by using *glyceraldehyde-3-phosphate dehydrogenase (GAPDH)* expression as an internal control for each sample as described elsewhere [36].

**Table 1:** Primers used in this study.

genes	upstream (5' to 3')	downstream (5' to 3')
GAPDH	AGGTCGGTGTGAACGGATTTG	TGTAGACCATGTAGTTGAGGTCA
slc30a1	TAACACCAGCAATTCCAACG	AGGACGTGCAGAAACACTCC
slc30a2	CTGCCTGGTGTTCATGATTG	CAAGGCTCCAAGGATCTCAG
slc30a3	GAAGAGTCTTTTCACAGAGCCC	TGTGTGCTAAATACCCACCAAC
slc30a4	TGCTGAGGAAAGACGACACG	GCCACCACGACTCGAAGTTTAT
slc30a5	GTGGAGCTAAGCGCCTTCAG	CCATAGCGGGCACATTTGG
slc30a6	TCCTGGCTGTATTTGCTTCTACT	CCAAAAAGCGTTCTGCACTTTC
slc30a7	CAGGCTGGTTTAGGTCCATCC	ATGCCGTAGAGTAGTTCCACA
slc30a8	TGATGCTGCTCATCTCTTAATTG	CTGCTCGATACCACCCAAATG
slc30a9	CATCCTCAACCAATGGAATCCC	TCATTTATGGCAACGAGAAGTGT
slc30a10	TCGAATGTAGCAGGTGATTCC	TCAAACTGGGGTCAATGTAGC
slc39a1	CTGCCATAGATGAGGCCTTG	TCCATCATGCCAATGTTGAG
slc39a2	GGGAGGGACTCATGCCTTTG	GTGGTCCAGTGCCGATCTTC
slc39a3	AGCGGCCTCCCTTTATAGAC	GGCTCTCGTACTCCGAGTC
slc39a4	ATGCTCCCAAAGTCGCTCAC	CAGCGTATTTAACAGGCCGTC
slc39a5	TATCGCATGGATGGTCCTC	CCTTCCTGAAGCAGCATTG
slc39a6	TTGATGCTCGGTCTTGTCTG	AGTGGCACCAAGATGACTCC
slc39a7	TGAATCTGGCTGCTGACTTG	GCAGTCAAGAGTTGCAGACG
slc39a8	CGATCCTGTGTGAGGAGTTTC	TCAGCATGTCGTTCATCTCTG
slc39a9	TGTTGGTGGGATGTTACGTGG	TGCTGCTTTGTCTGATGCAAT
slc39a10	CATAATCGGGTTCACAAACTTGA	GCTTCTCGCTTTCGAGTATGTCG
slc39a11	ACAAGCGTGAGAATGGCGAG	TGGCAGATGCAGTCTTTTCTAC
slc39a12	GACATCTTGGCTTCCACCAG	CAAACTCCTTGGAGCGACAG
slc39a13	CCTGGCTGTGGTATGGCAG	ACTGAGCCCAACCATGAGAGA
slc39a14	CCTCAGGACAATTATGTCTCCA	ATGGTGCTCGTTTTTCTGCTT

### Data Analysis

Results are all expressed as the mean ± S.E. and the statistical significance was determined by the one-way or two-way ANOVA with Bonferroni/Dunnett *post hoc* test, or the two-tailed Students’ t-test. The level of significance was set at *p*<0.05.

## Results

### An increase by Zn^2+^ in Fluo-3 fluorescence in C6 glioma cells

**Fig 1:**
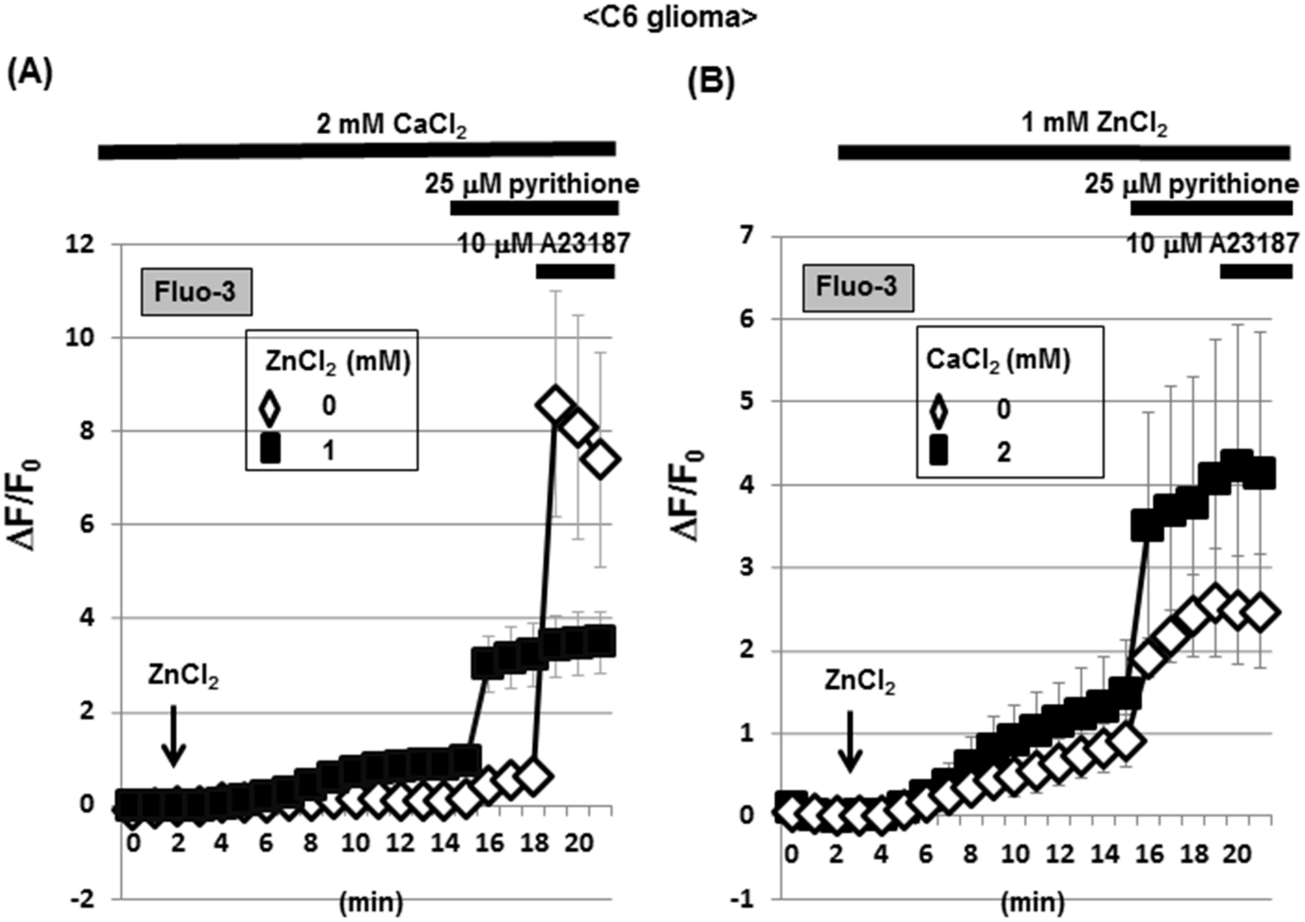
Effects of ZnCl_2_ on Fluo-3 fluorescence in C6 glioma cells. (A) C6 glioma cells were cultured for 24 h, followed by loading of Fluo-3 in the presence of CaCl_**2**_ and subsequent determination of the fluorescence intensity in either the presence or absence of ZnCl_**2**_ every 1 min for 21 min. (B) Cells were loaded with Fluo-3 in either the presence or absence of CaCl_**2**_, followed by determination of the fluorescence intensity in the presence of ZnCl_**2**_ every 1 min. Values are the mean±S.E. of the rate of fluorescence change in 3 different experiments.

**Fig 2:**
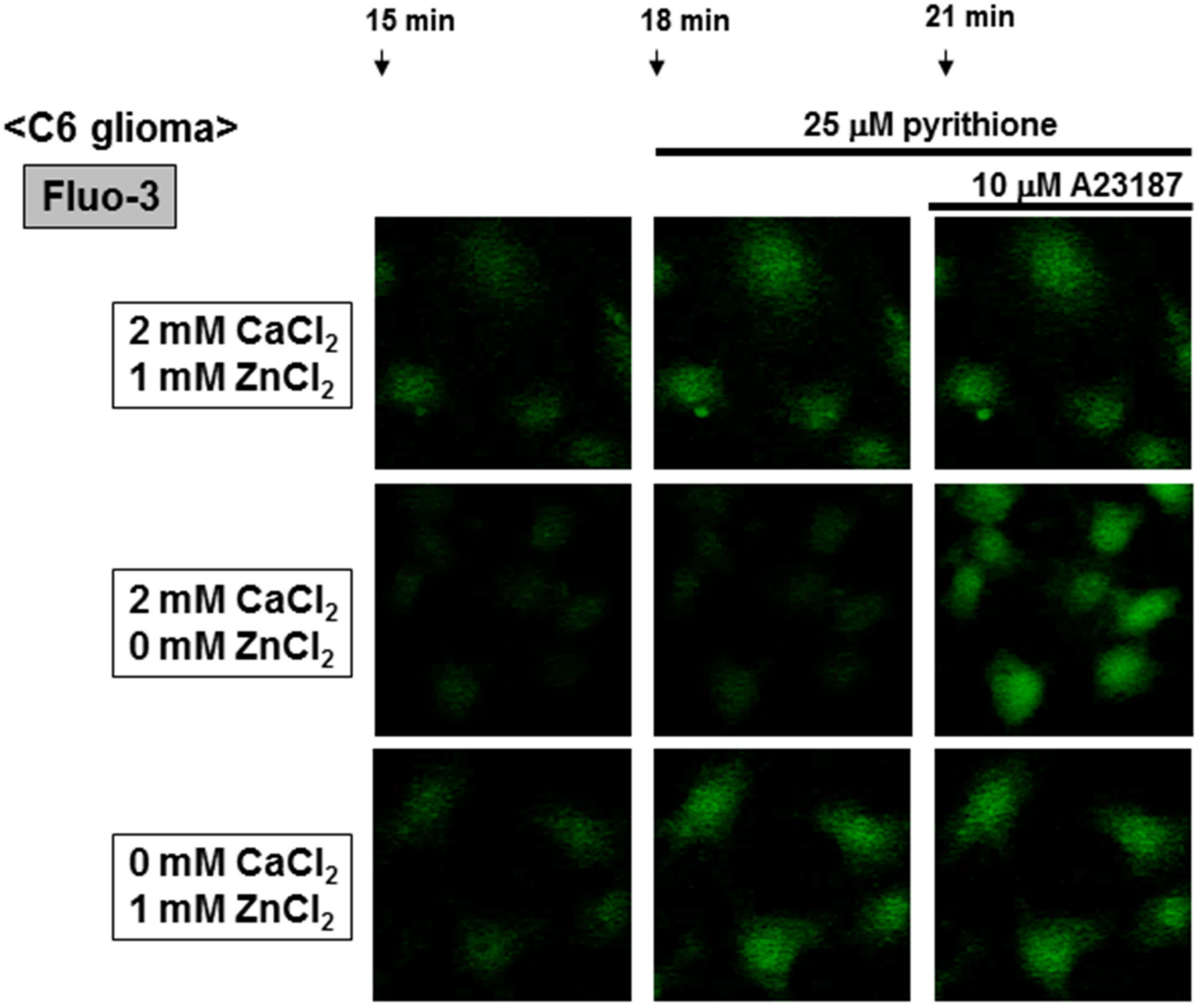
Micrographic pictures of Fluo-3 fluorescence in C6 glioma cells exposed to CaCl_2_ and ZnCl_2_ in the presence of A23187 and pyrithione. Typical pictures are shown here.

Rat C6 glioma cells were loaded with Fluo-3, followed by addition of 2 mM CaCl_2_ in either the presence or absence of 1 mM ZnCl_2_ and subsequent determination of the fluorescence intensity every 1 min. Exposure to ZnCl_2_ led to a gradual and spontaneous increase in the fluorescence intensity of Fluo-3 in the presence of CaCl_2_ throughout, while the zinc ionophore pyrithione markedly increased the Fluo-3 fluorescence in the presence of ZnCl_2_ without affecting that in the absence of ZnCl_2_ (Figs 1A and 2). Subsequent further addition of the calcium ionophore A23187 failed to additionally increase the fluorescence already elevated by pyrithione in the presence of ZnCl_2_, but drastically increased the fluorescence in the absence of ZnCl_2_. In the presence of ZnCl_2_ throughout, in contrast, a gradual increase was seen in Fluo-3 fluorescence with a drastic increase by pyrithione in a manner irrespective of the addition of CaCl_2_ (Fig 1B). Subsequent further addition of A23187 again failed to affect Fluo-3 fluorescence independent of the presence of CaCl_2_.

**Fig 3:**
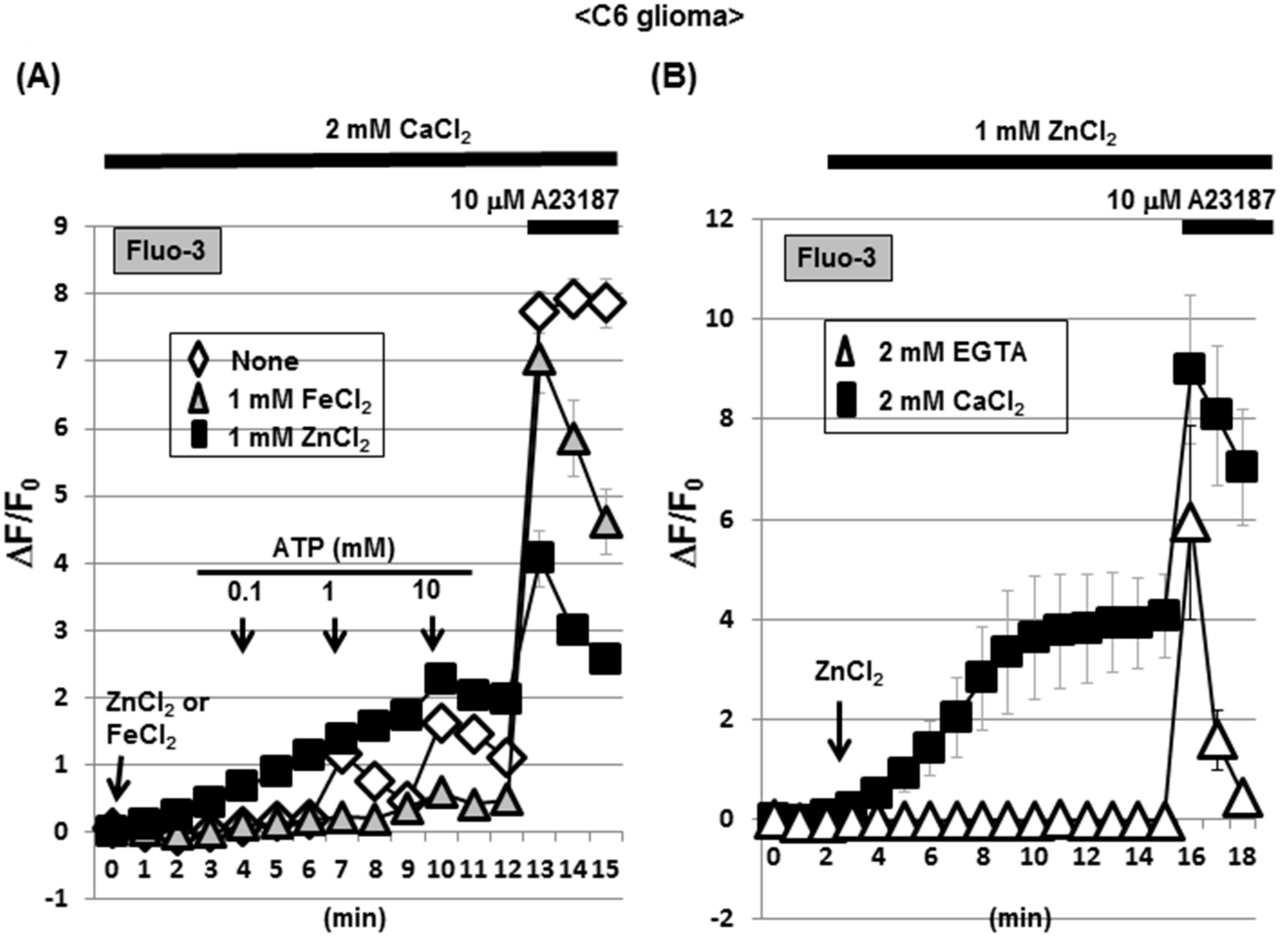
A selective increase by ZnCl_2_ in Fluo-3 fluorescence in C6 glioma cells. (A) Cells were loaded with Fluo-3 in the presence of CaCl_**2**_, followed by determination of the fluorescence intensity in either the presence or absence of ZnCl_**2**_ and FeCl_**2**_ every 1 min. Cells were exposed to ATP at different concentrations during the determination of fluorescence. (B) Cells were loaded with Fluo-3 in the presence of either EGTA or CaCl_**2**_, followed by determination of the fluorescence intensity in the presence of ZnCl_**2**_ every 1 min. Values are the mean±S.E. of the rate of fluorescence change in 3 different experiments.

**Fig 4:**
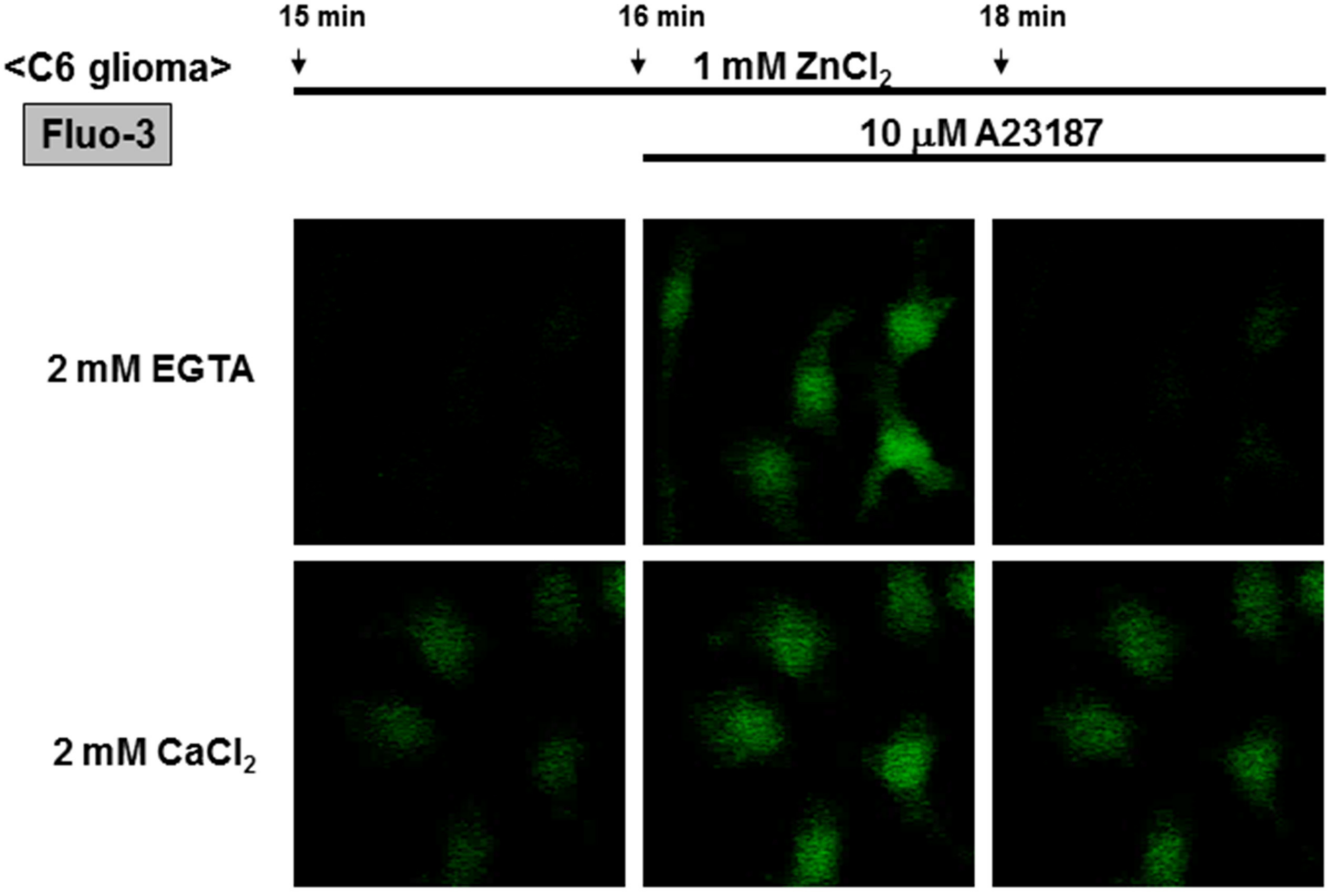
Micrographic pictures of Fluo-3 fluorescence in C6 glioma cells exposed to CaCl_2_ and ZnCl_2_ in the presence of A23187 and EGTA. Typical pictures are shown here.

The addition of ATP did not markedly affect Fluo-3 fluorescence in C6 glioma cells known to constitutively express different ionotropic P2X receptor subtypes permeable for Ca^2+^ [37], while Fluo-3 fluorescence was again gradually increased in cells exposed to ZnCl_2_, but not to FeCl_2_, in the presence of CaCl_2_ (Fig 3A). Further addition of A23187 invariably increased Fluo-3 fluorescence irrespective of the exposure to FeCl_2_ and ZnCl_2_. In the presence of ZnCl_2_ throughout, further addition of CaCl_2_ led to a gradual increase in Fluo-3 fluorescence in a manner sensitive to the calcium chelator EGTA (Figs 3B and 4). In the presence of EGTA, however, A23187 induced a transient increase in Fluo-3 fluorescence along with a rapid decline to the basal level.

### An increase by Zn^2+^ in Rhod-2 and FluoZin-3 fluorescence in C6 glioma cells

**Fig 5:**
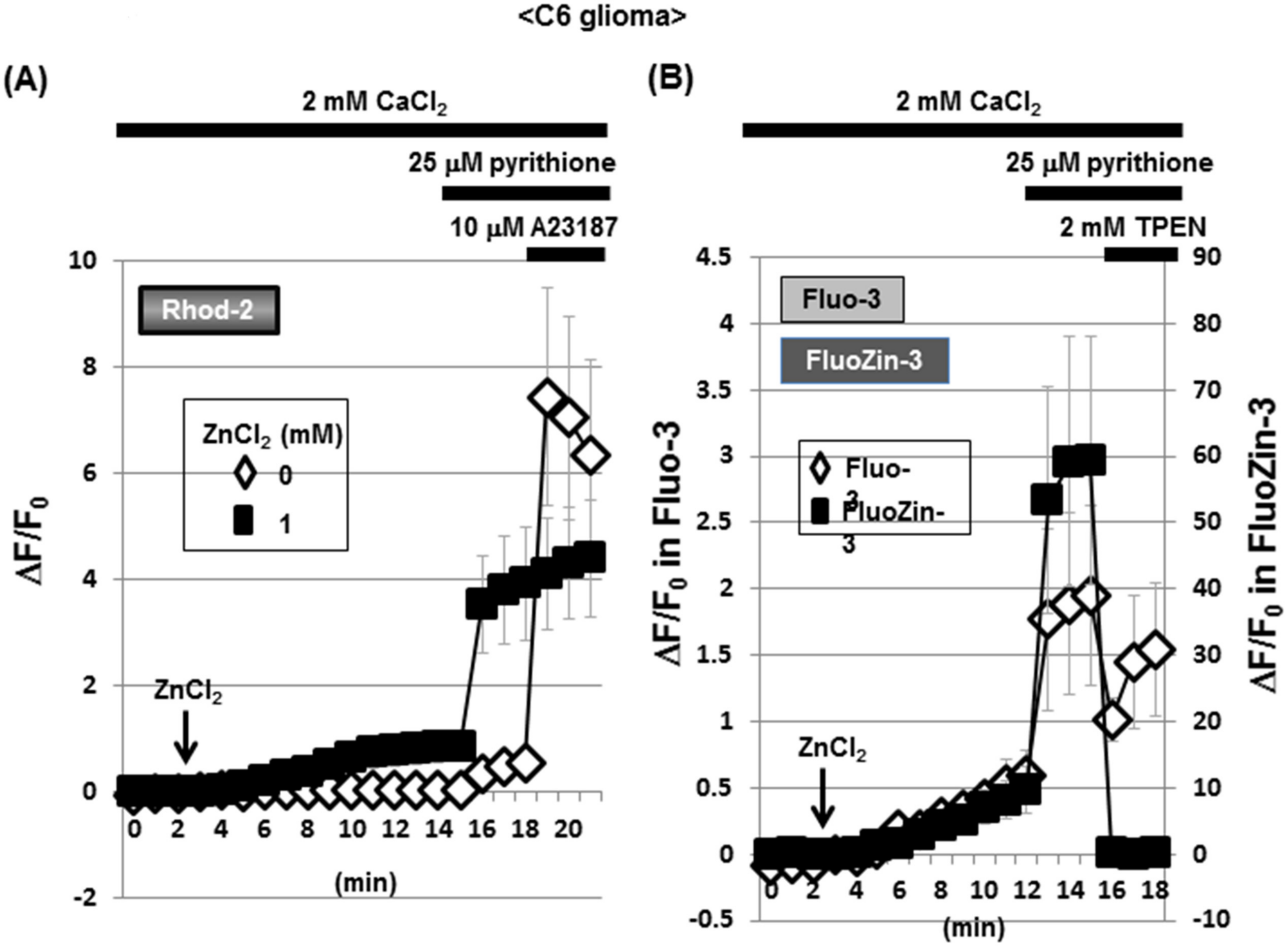
Possible interaction of Ca^2+^-sensitive dyes with Zn^2+^ in C6 glioma cells. (A) C6 glioma cells were loaded with Rhod-2 in the presence of CaCl_**2**_, followed by determination of the fluorescence intensity in either the presence or absence of ZnCl_**2**_ every 1 min. (B) Cells were loaded with either Fluo-3 or FluoZin-3 in the presence of CaCl_**2**_, followed by determination of the fluorescence intensity in the presence of ZnCl_**2**_ every 1 min. Values are the mean±S.E. of the rate of fluorescence change in 3 different experiments.

**Fig 6:**
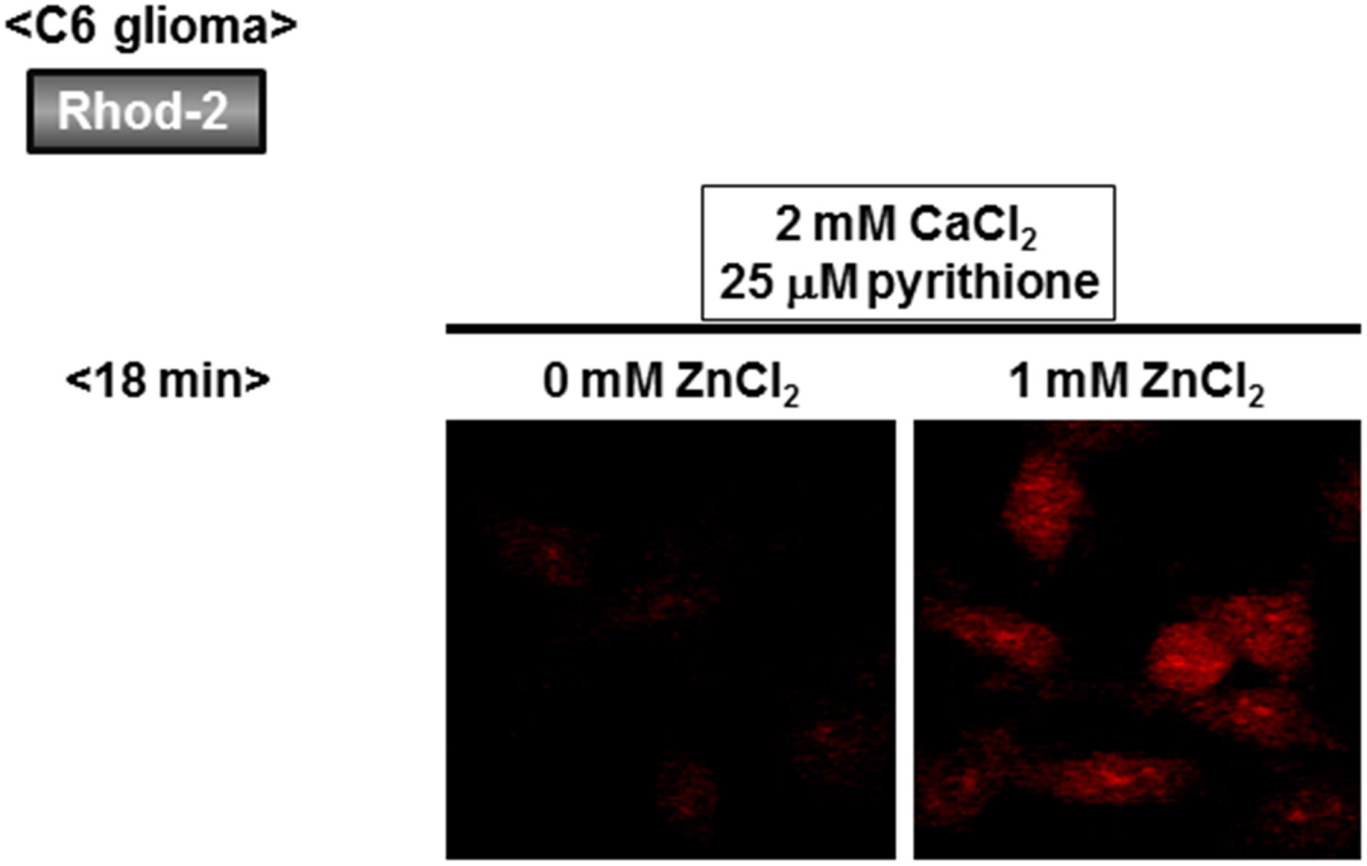
Micrographic pictures of Rhod-2 fluorescence in C6 glioma cells exposed to CaCl_2_ and ZnCl_2_ in the presence of pyrithione. Typical pictures are shown here.

**Fig 7:**
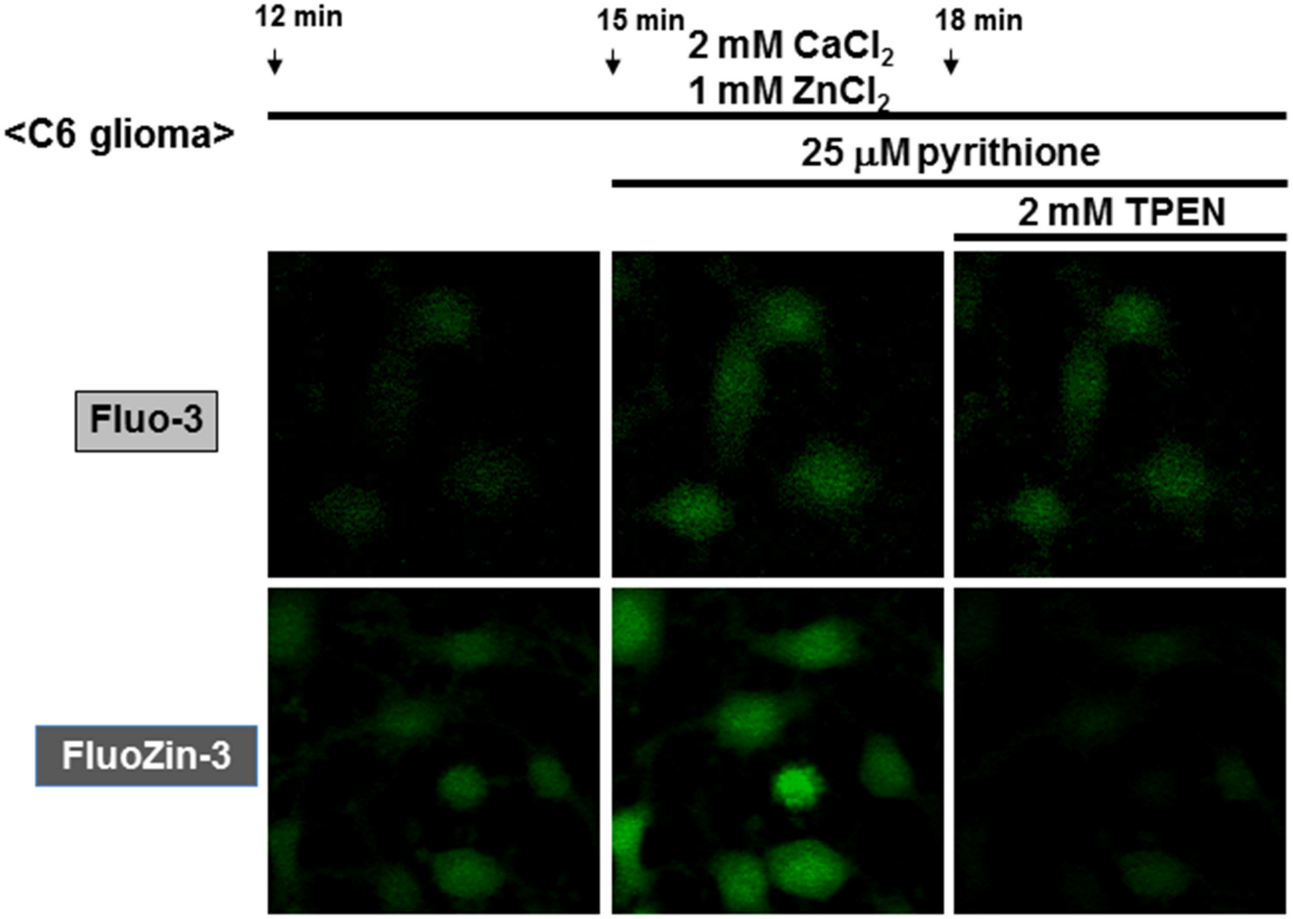
Micrographic pictures of both Fluo-3 and FluoZin-3 fluorescence in C6 glioma cells exposed to CaCl_2_ and ZnCl_2_ in the presence of pyrithione and TPEN. Typical pictures are shown here.

The Ca^2+^-sensitive fluorescent dye Rhod-2 is known to be accumulated into mitochondria due to its high cationic charge, with the fluorescence being predominantly detected in intracellular areas merged with MitoTracker fluorescence [10]. C6 glioma cells were thus loaded with Rhod-2, followed by exposure to CaCl_2_ in either the presence or absence of ZnCl_2_ and subsequent addition of pyrithione and A23187. In the absence of ZnCl_2_, A23187 markedly increased Rhod-2 fluorescence with pyrithione being ineffective in the presence of 2 mM CaCl_2_ throughout (Figs 5A and 6). In the presence of both ZnCl_2_ and CaCl_2_, in contrast, a spontaneous gradual increase was seen in Rhod-2 fluorescence with a drastic increase by pyrithione in a manner irrespective of the addition of A23187. We next used the Zn^2+^-sensitive fluorescent dye FluoZin-3 for monitoring intracellular Zn^2+^ levels, in addition to Fluo-3, in cultured glioma cells. In the presence of both CaCl_2_ and ZnCl_2_, a spontaneous gradual increase was similarly seen in the fluorescence of both fluorescent dyes (Figs 5B and 7). Although pyrithione similarly induced a drastic increase in both Fluo-3 and FluoZin-3 fluorescence, the Zn^2+^ chelator TPEN was highly effective in inhibiting FluoZin-3 fluorescence without affecting Fluo-3 fluorescence.

### A selective increase by Zn^2+^ in FluoZin-3 fluorescence in C6 glioma cells

**Fig 8:**
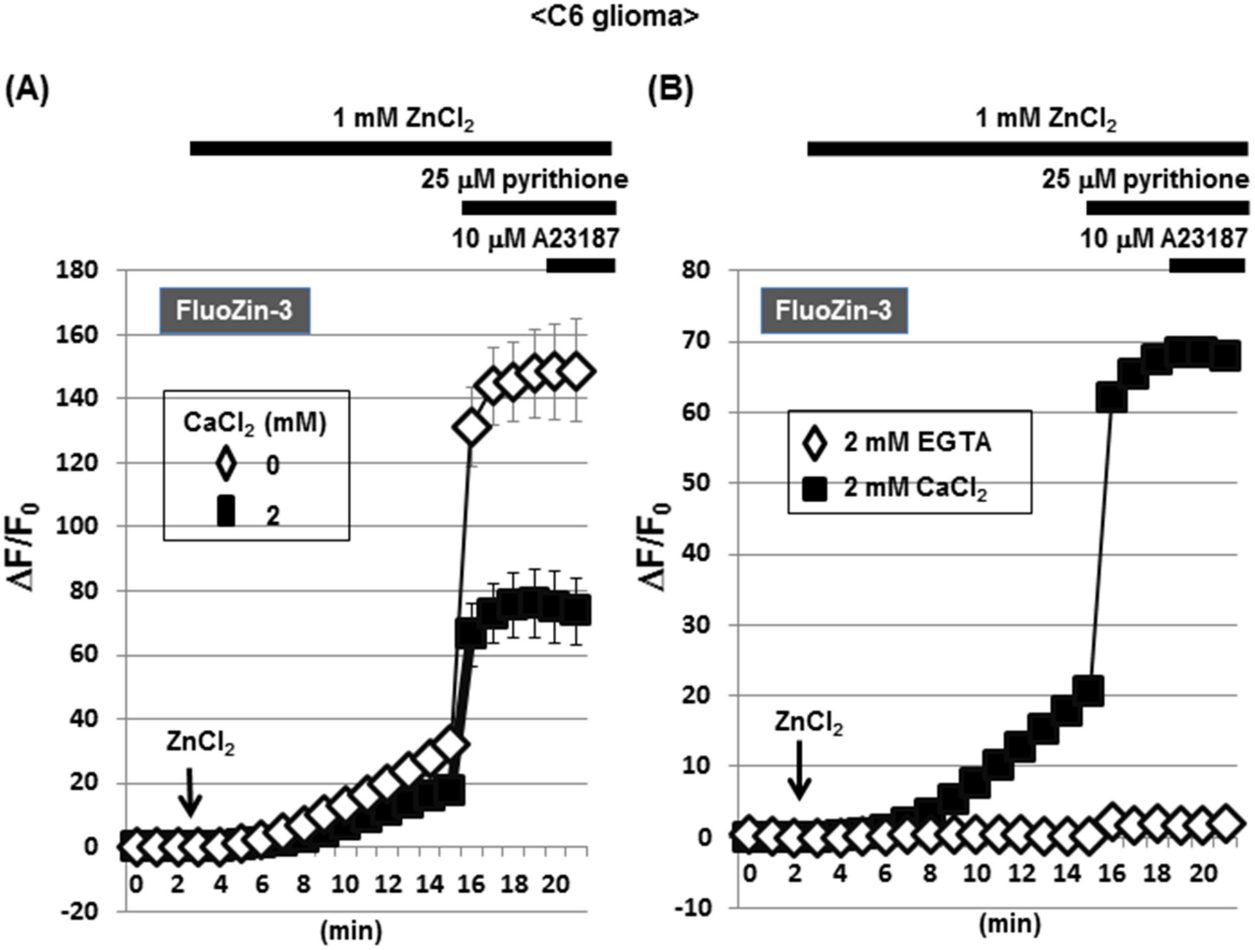
Effects of ZnCl_2_ on FluoZin-3 fluorescence in C6 glioma cells. (A) C6 glioma cells were loaded with FluoZin-3 in either the presence or absence of CaCl_**2**_, followed by determination of the fluorescence intensity in the presence of ZnCl_**2**_ every 1 min. (B) Cells were loaded with FluoZin-3 in the presence of either EGTA or CaCl_**2**_, followed by determination of the fluorescence intensity in the presence of ZnCl_**2**_ every 1 min. Values are the mean±S.E. of the rate of fluorescence change in 3 different experiments.

**Fig 9:**
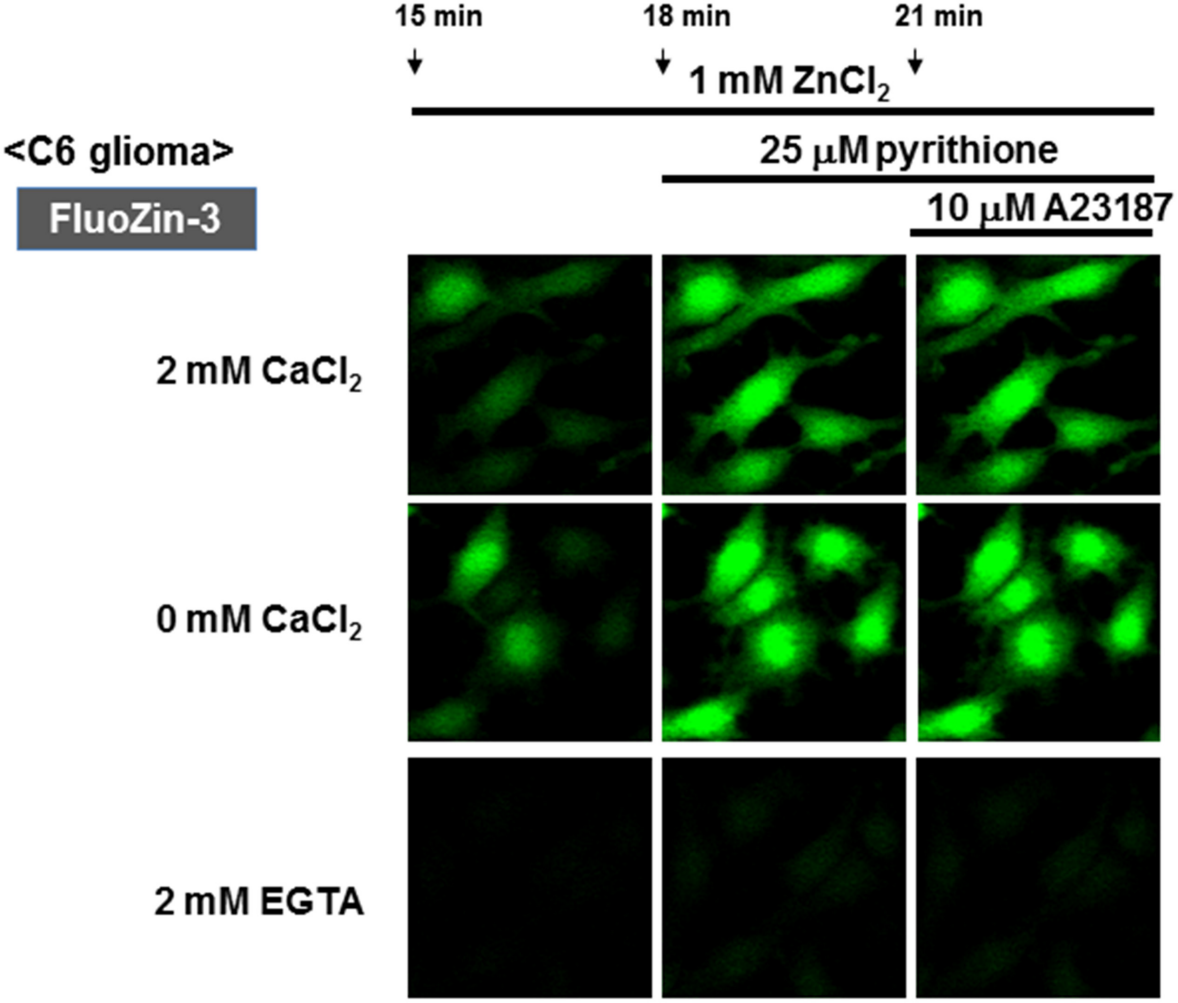
Micrographic pictures of FluoZin-3 fluorescence in C6 glioma cells exposed to CaCl_2_ and ZnCl_2_ in the presence of A23187 and pyrithione. Typical pictures are shown here.

Glioma cells were loaded with FluoZin-3, followed by exposure to ZnCl_2_ in either the presence or absence of CaCl_2_. Irrespective of the addition of CaCl_2_, a spontaneous gradual increase was invariably seen in FluoZin-3 fluorescence along with a drastic increase by pyrithione (Figs 8A and 9). Further addition of A23187 failed to additionally increase FluoZin-3 fluorescence elevated by pyrithione irrespective of the addition of CaCl_2_. In the presence of EGTA, however, exposure to ZnCl_2_ did not induce a spontaneous gradual increase in FluoZin-3 fluorescence with both pyrithione and A23187 being ineffective (Fig 8B).

### Artificial NMDAR channels permeable for Ca^2+^ in HEK293 cells

**Fig 10:**
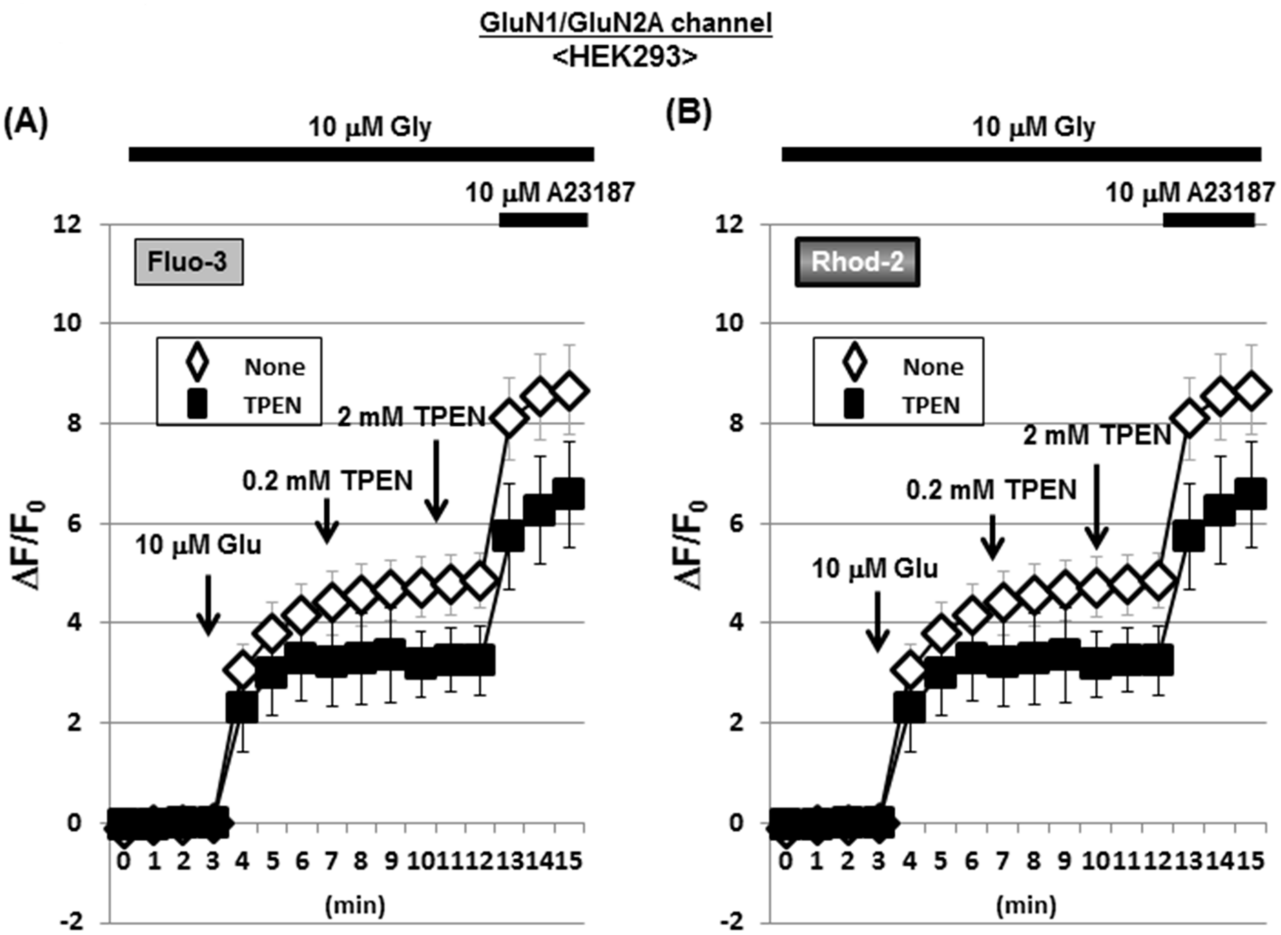
Effects of TPEN on Fluo-3 and Rhod-2 fluorescence in HEK293 cells with acquired NMDAR channels. HEK293 cells were transfected with expression vectors of GluNR1 and GluNR2A, followed by further culture for an additional 24 h and subsequent loading of either (A) Fluo-3 or (B) Rhod-2 in the presence of Gly. Cells were then exposed to Glu in either the presence or absence of TPEN during the determination of each fluorescence intensity every 1 min. Values are the mean±S.E. of the rate of fluorescence change in 3 different experiments.

**Fig 11:**
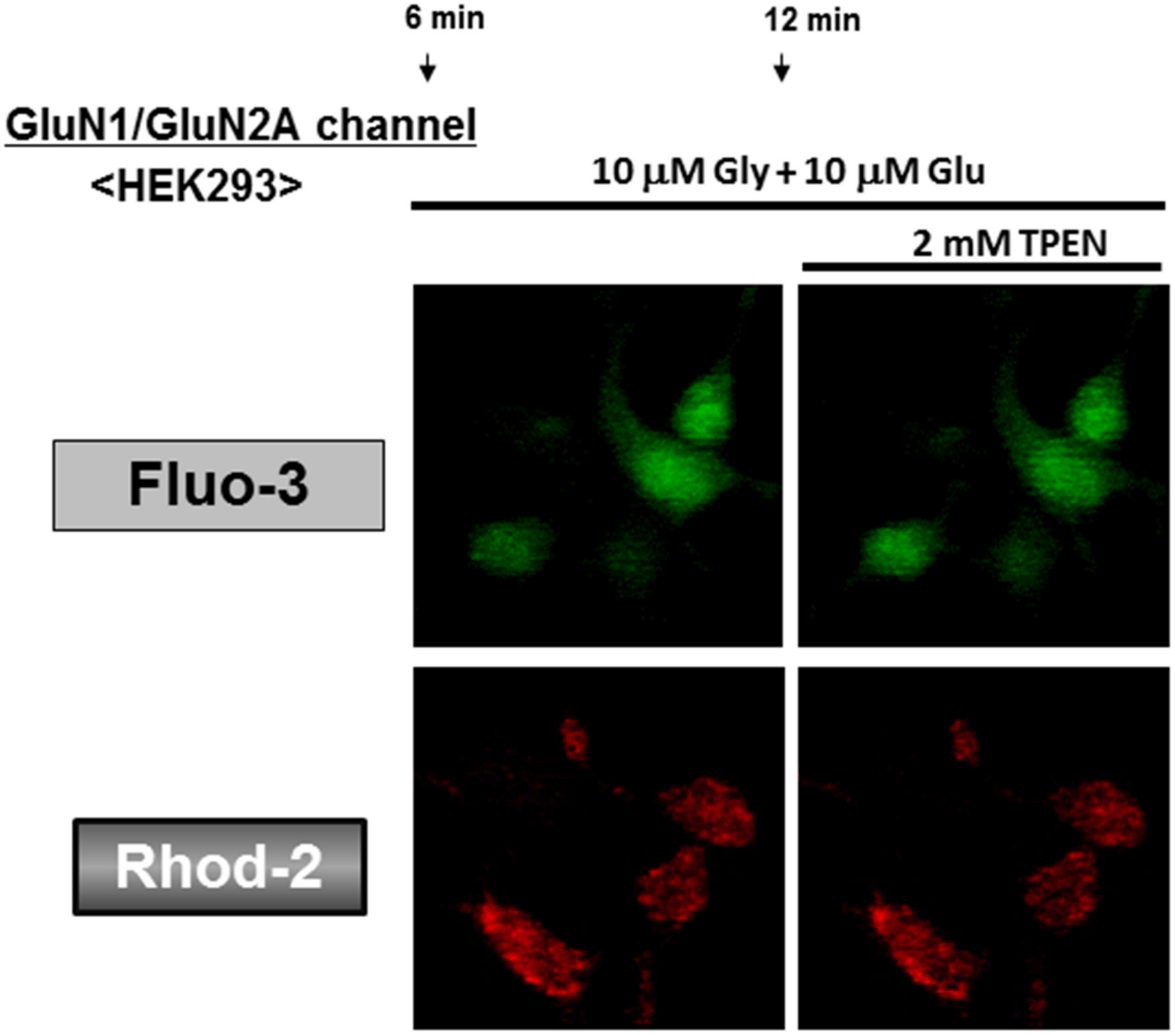
Micrographic pictures of both Fluo-3 and FluoZin-3 fluorescence in HEK293 cells with artificial NMDAR. Typical pictures are shown here.

To test the selectivity of the zinc chelator TPEN for Zn^2+^, we artificially orchestrated acquired NMDAR channels highly permeable for Ca^2+^ in HEK293 cells devoid of any NMDAR subunits [33]. As both Glu and glycine (Gly) are inevitably required for the opening of NMDAR channels, HEK293 cells were transfected with both GluN1 and GluN2A subunit expression vectors, followed by loading of both Fluo-3 and Rhod-2, and subsequent exposure to Glu in the presence of Gly. The addition of Gly alone did not markedly increase the fluorescence of Fluo-3 (Fig 10A) and Rhod-2 (Fig 10B) in cells with artificial NMDAR channels, while further addition of Glu markedly increased both Fluo-3 and Rhod-2 fluorescence in a manner insensitive to TPEN at concentrations of 0.2 and 2 mM (Fig 11). Consequential further addition of A23187 drastically increased both Fluo-3 and Rhod-2 fluorescence in the presence of both Glu and Gly independent of the addition of TPEN.

### Inhibition by Zn^2+^ of cellular vitality in C6 glioma cells

**Fig 12:**
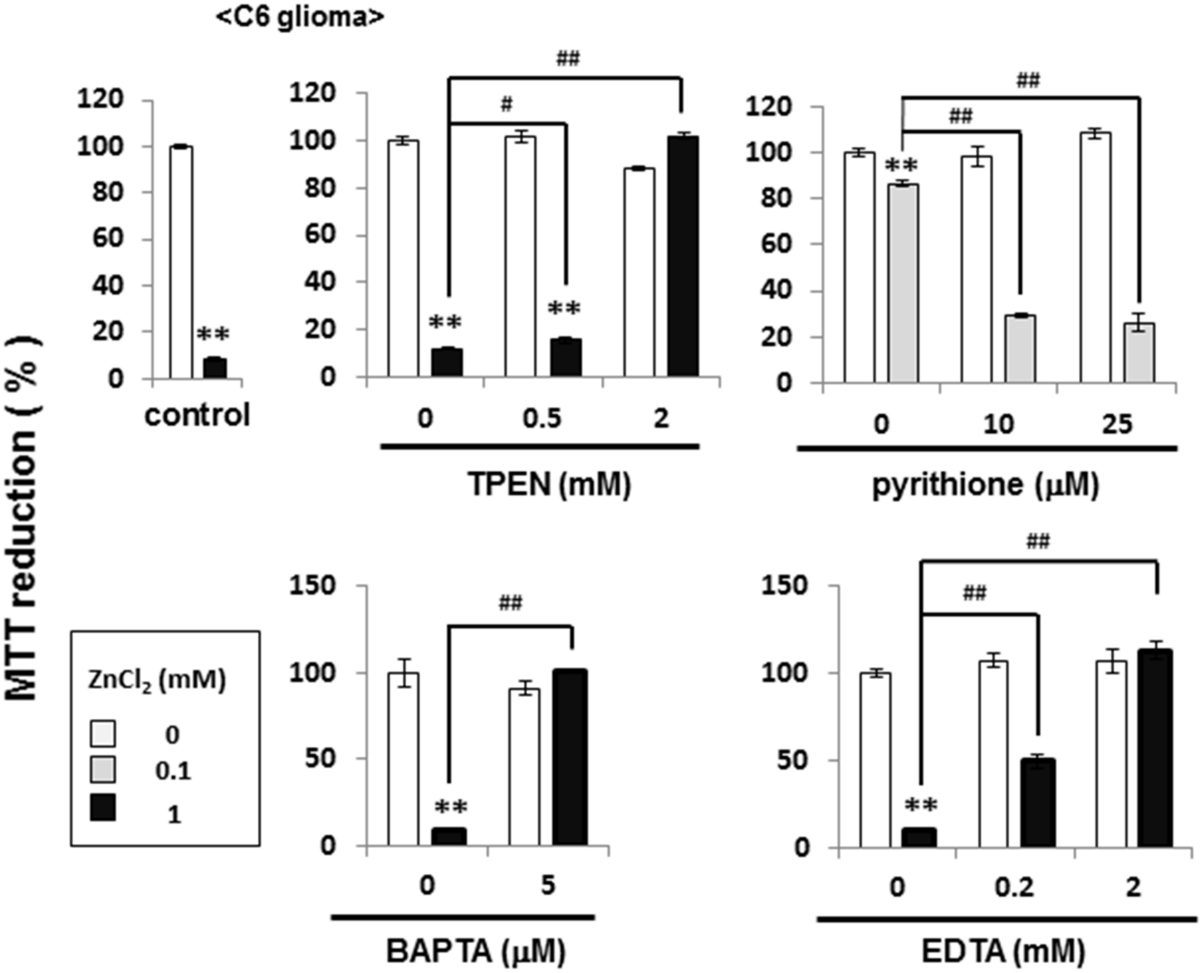
Effects of ZnCl_2_ on MTT reducing activity in C6 glioma cells. Cells were exposed to ZnCl_**2**_ at different concentrations in either the presence or absence of TPEN, pyrithione, BAPTA and EDTA for 1 h, followed by culture for an additional 6 h and subsequent determination of MTT reducing activity. Values are the mean±S.E. of percentages over the maximal activity detected in cells not exposed to any test chemicals in 3 different experiments. *P<0.05, **P<0.01, significantly different from the control value in cells not exposed to ZnCl_2_. ^#^P<0.05, ^#^P<0.01, significantly different from the value in cells exposed to ZnCL_2_ at each concentration.

C6 glioma cells were exposed to ZnCl_2_ for 1 h, followed by further culture for an additional 24 h and subsequent determination of MTT reducing activity as an index of cellular vitality. Exposure to 1 mM ZnCl_2_ led to more than 80% inhibition of MTT reduction (Fig 12, upper left panel), while TPEN significantly prevented the inhibition by 1 mM ZnCl_2_ in a concentration-dependent manner at concentrations of 0.5 to 2 mM (Fig 12, upper middle panel). In contrast, pyrithione was effective at concentrations of 10 to 25 μM in significantly exacerbating the inhibition by 0.1 mM ZnCl_2_ of MTT reduction (Fig 12, upper right panel). In a manner similar to the Zn^2+^ chelator TPEN, a significant prevention was seen for the inhibition by 1mM ZnCl_2_ in the presence of BAPTA (Fig 12, lower left panel) and EDTA (Fig 12, lower right panel), which have been used as intracellular and extracellular Ca^2+^ chelators for years, respectively.

**Fig 13:**
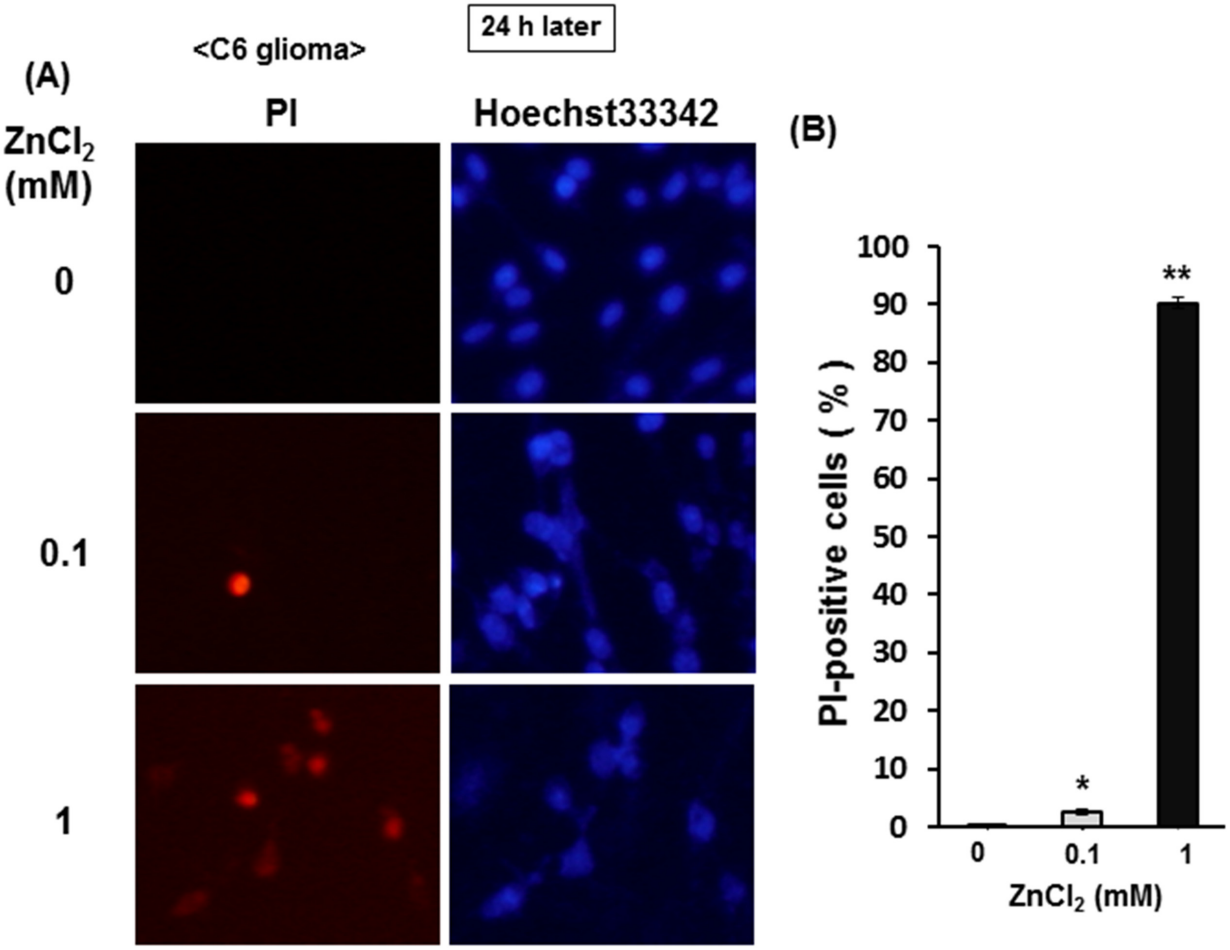
Effects of ZnCl_2_ on PI and Hoechst33342 staining in C6 glioma cells. Cells were exposed to ZnCl_**2**_ at concentrations of 0.1 to 1 mM in the presence of CaCl_**2**_ for 1 h, followed by culture for an additional 24 h and subsequent double staining with PI and Hoechst33342 for nuclear DNA. Typical micrographs are shown in the panel (A), while in the panel (B) value are the mean±S.E. of percentages of PI-positive cells over Hoechst33342-positive cells in 3 independent experiments. *P<0.05, **P<0.01, significantly different from the control value in cells not exposed to ZnCl_**2**_.

The percentage of injured cells stained with the membrane-impermeable DNA dye PI was markedly increased over the total cells stained with membrane-permeable DNA dye Hoechst33342 in cells exposed to ZnCl_2_ in a concentration-dependent manner, whereas the number of cell stained with Hoechst33342 was drastically decreased in cultured cells previously exposed to 1 mM ZnCl_2_ for 1 h when observed 24 h after exposure (Fig 13A). Quantitative analysis clearly revealed that more than 90% of cells were stained with PI in C6 glioma cells exposed to 1 mM ZnCl_2_ for 1 h when calculated 24 h later (Fig 13B).

**Fig 14:**
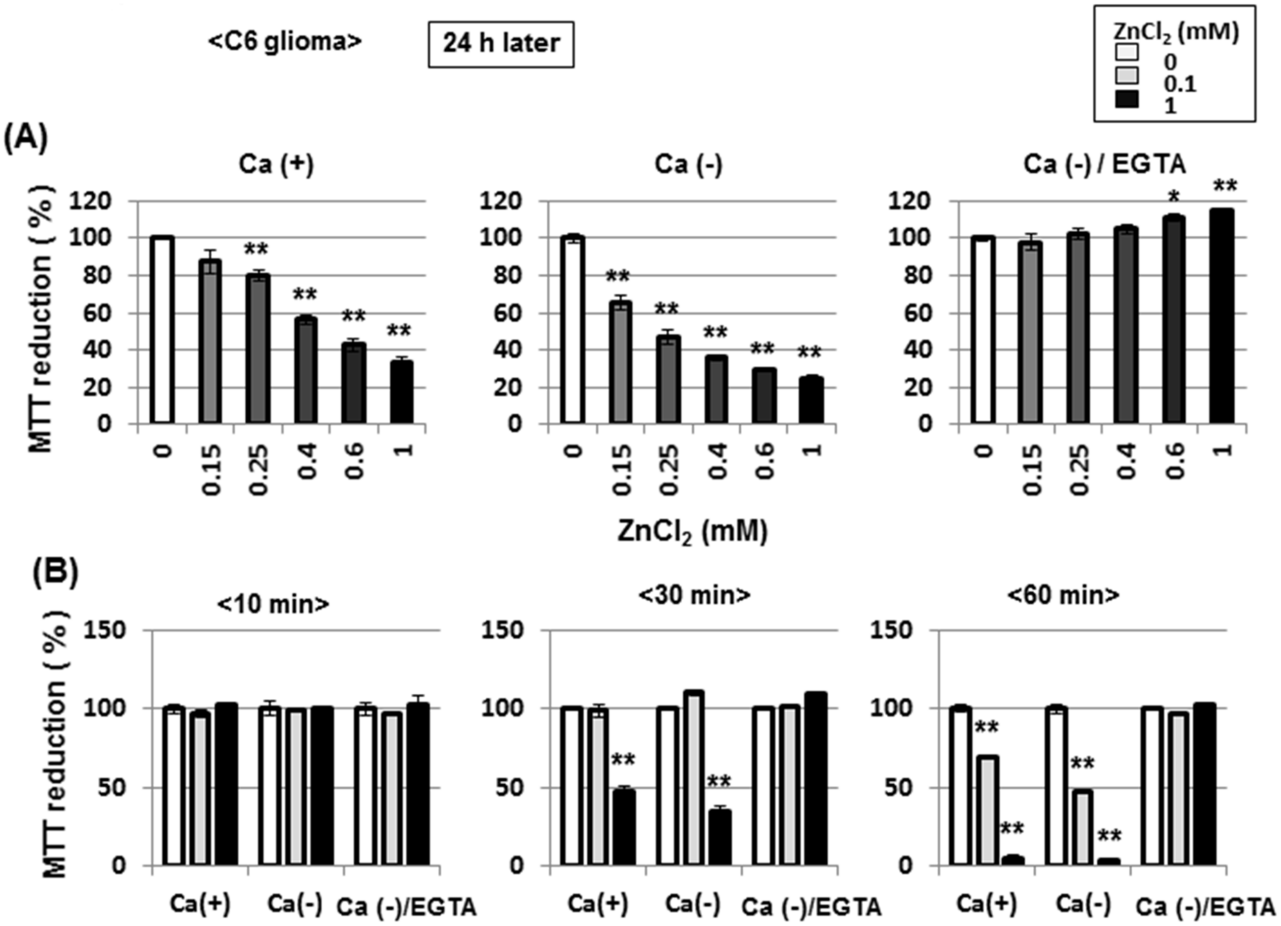
Effects of EGTA on ZnCl_2_-induced inhibition of MTT reducing activity in C6 glioma cells. (A) Cells were exposed to ZnCl_**2**_ at concentrations of 0.15 to 1 mM in either the presence or absence of CaCl_**2**_ and EGTA for 1 h, followed by culture for an additional 24 h and subsequent determination of MTT reducing activity. (B) Cells were also exposed to ZnCl_**2**_ at 0.1 or 1 mM in either the presence or absence of CaCl_**2**_ and EGTA for different periods from 10 to 60 min, followed by culture for an additional 24 h and subsequent determination of MTT reducing activity. Values are the mean±S.E. of percentages over the maximal activity detected in cells not exposed to any test chemicals in 3 different experiments. *P<0.05, **P<0.01, significantly different from the control value in cells not exposed to ZnCl_**2**_.

Cells were next exposed to ZnCl_2_ at different concentrations of 0.15 to 1 mM for 60 min in either the presence or absence of CaCl_2_ and EGTA, followed by further culture for an additional 24 h and subsequent determination of MTT reducing activity. Exposure to ZnCl_2_ for 60 min induced a concentration-dependent inhibition of MTT reduction determined 24 h later in a fashion irrespective of the addition of CaCl_2_, whereas further addition of 2 mM EGTA completely prevented the inhibition by ZnCl_2_ in the absence of CaCl_2_ (Fig 14A). Prior exposure to ZnCl_2_ for 10 min did not significantly affect MTT reduction independent of the addition of CaCl_2_ and EGTA when determined 24 h after exposure, while a significant inhibition of MTT reduction was found in cells exposed to ZnCl_2_ for 30 to 60 min irrespective of the addition of CaCl_2_ (Fig 14B). In cells with further addition of EGTA along with removal of CaCl_2_, ZnCl_2_ failed to significantly inhibit MTT reduction at concentrations of 0.1 to 1 mM.

**Fig 15:**
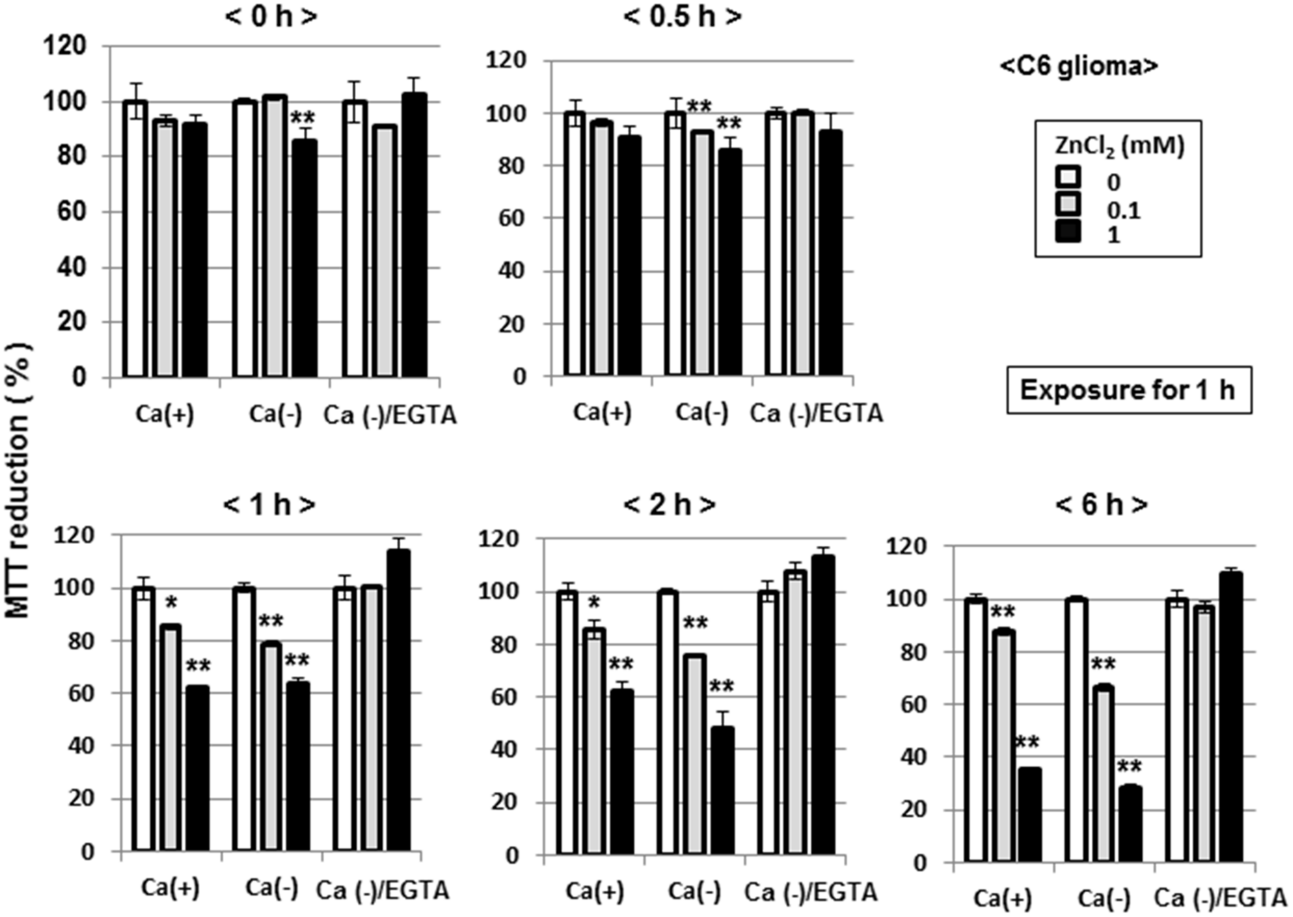
Effects of culture periods on ZnCl_2_-induced inhibition of MTT reducing activity in C6 glioma cells. Cells were exposed to ZnCl_**2**_ at 0.1 or 1 mM in either the presence or absence of CaCl_**2**_ and EGTA for 1 h, followed by culture for an additional periods from 0.5 to 6 h and subsequent determination of MTT reducing activity. Values are the mean±S.E. of percentages over the maximal activity detected in cells not exposed to any test chemicals in 3 different experiments. *P<0.05, **P<0.01, significantly different from the control value in cells not exposed to ZnCl_**2**_.

C6 glioma cells were exposed to ZnCl_2_ at 0.1 or 1 mM for 1 h, followed by further culture for an additional period of up to 6 h and subsequent determination of MTT reduction. No significant inhibition of MTT reduction was seen in cells collected immediately (Fig 15, upper left panel) and 30 min (Fig 15, upper right panel) after the exposure to ZnCl_2_ for 1 h in a manner independent of the addition of CaCl_2_ and EGTA. In cells collected 1 (Fig 15, lower left panel), 2 (Fig 15, lower middle panel) and 6 h (Fig 15, lower right panel) after the exposure to ZnCl_2_ for 1 h, by contrast, a significant inhibition of MTT reduction was induced in a fashion irrespective of the addition of CaCl_2_, but in a manner prevented by EGTA.

### Inhibition by Zn^2+^ of cellular vitality in other cell lines

**Fig 16:**
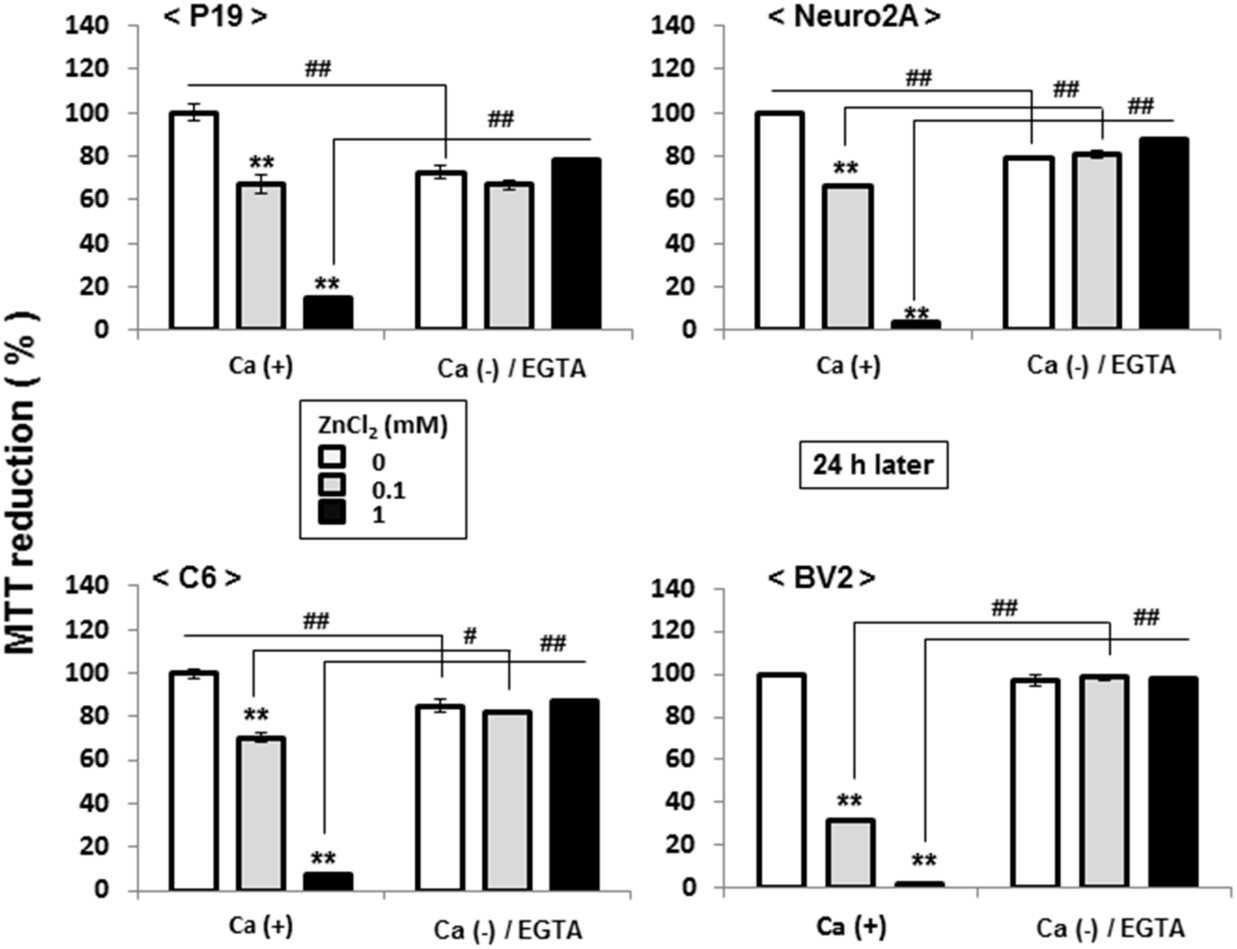
Effects of ZnCl_2_ on MTT reducing activity in different cell lines. Cells were exposed to ZnCl_**2**_ at 0.1 or 1 mM in either the presence or absence of CaCl_**2**_ and EGTA for 1 h, followed by culture for an additional 24 h and subsequent determination of MTT reducing activity. Values are the mean±S.E. of percentages over the maximal activity detected in cells not exposed to any test chemicals in 3 different experiments. *P<0.05, **P<0.01, significantly different from the control value in cells not exposed to ZnCl_2_. ^#^P<0.05, ^#^P<0.01, significantly different from the value in cells exposed to ZnCL_2_ at each concentration.

In addition to astrocytic C6 glioma cells, pluripotent P19 cells, neuronal Neuro2A cells and microglial BV2 cells were individually exposed to ZnCl_2_ at 0.1 or 1 mM for 1 h in either the presence or absence of CaCl_2_ and EGTA, followed by further culture for an additional 24 h and subsequent determination of MTT reduction. In these parallel experiments, ZnCl_2_ was invariably effective in significantly inhibiting MTT reduction in the presence of CaCl_2_ in a concentration-dependent manner in P19 (Fig 10, upper left panel), Neuro2A (Fig 16, upper right panel), C6 (Fig 16, lower left panel) and BV2 (Fig 16, lower right panel) cells, which was never seen in cultured cells additionally exposed to EGTA in the absence of CaCl_2_ irrespective of the types of cell lines used.

### Expression profiles of Zn^2+^ transporters in different cell lines

**Fig 17:**
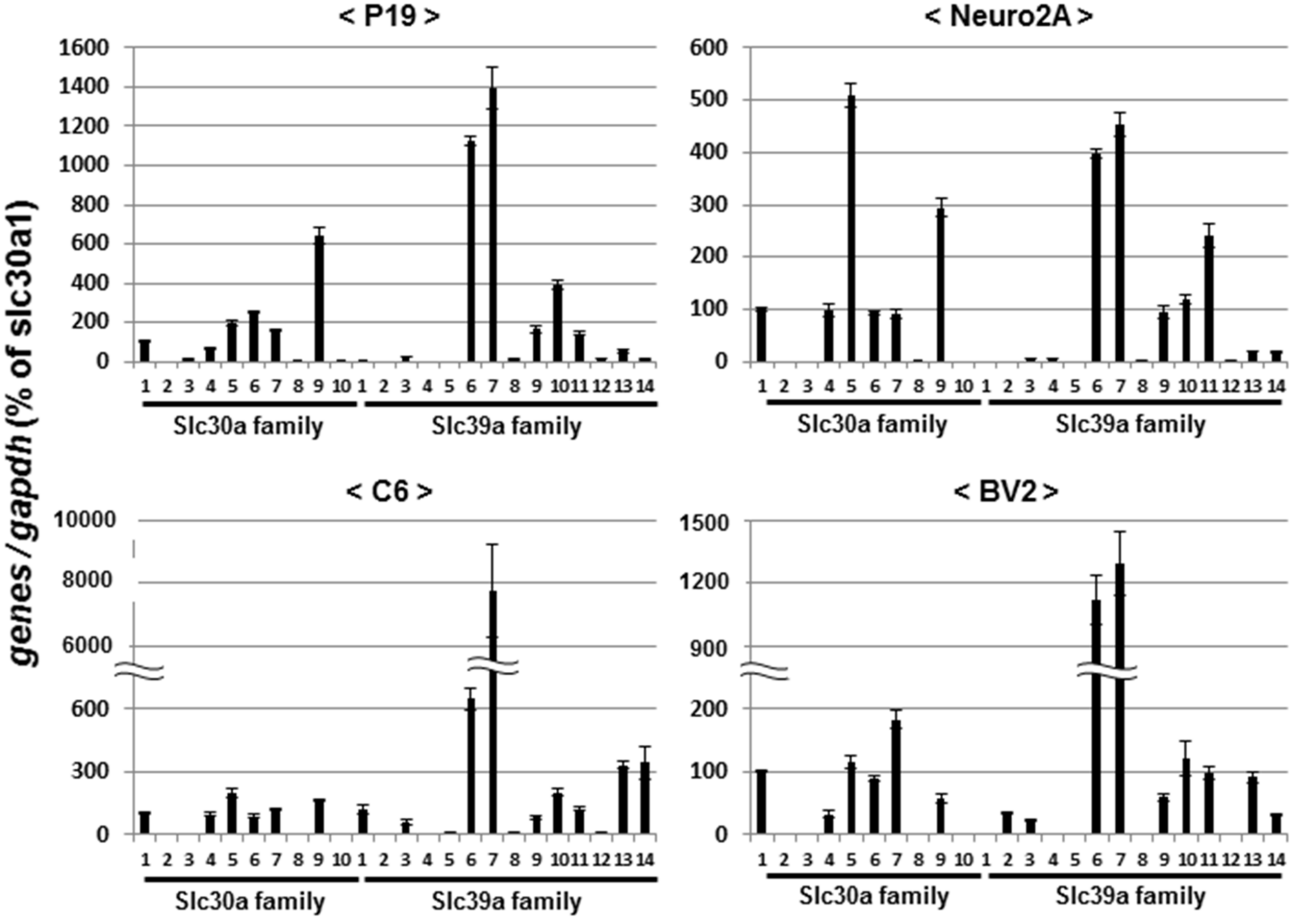
Expression profiles of Zn^2+^ transporters in different cell lines. Cells were cultured under respective appropriate conditions, followed by extraction of total RNA and subsequent determination of mRNA expression on qPCR. Values are the mean±S.E. of percentages over the expression of *Slc30a1* in 3 different experiments.

As cellular Zn^2+^ homeostasis is shown to at least in part involve bidirectional transport mediated by member proteins of the Zn^2+^ exporter solute carrier 30 (SLC30) family and the Zn^2+^ importer SLC39 family across membranes [38], we evaluated the possible expression of mRNA for these transmembrane Zn^2+^ transporters by different cell lines sensitive to the cytotoxicity of ZnCl_2_. Real time qPCR analysis clearly revealed constitutive mRNA expression of a variety of members of both *Slc30a* and *Slc39a* families in P19 (Fig 11, upper left panel), Neuro2A (Fig 17, upper right panel), C6 (Fig 17, lower left panel) and BV2 (Fig 17, lower right panel) cells. In particular, high expression of both *Slc39a6* and *Slc39a7* was commonly found in all cell lines tested which were sensitive to the cytotoxicity of ZnCl_2_.

## Discussion

The essential importance of the present findings is that exposure to ZnCl_2_ induced a spontaneous gradual increase in Fluo-3 fluorescence in an EGTA-sensitive manner irrespective of the presence of added CaCl_2_ in C6 glioma cells. Moreover, both A23187 and pyrithione were similarly effective in drastically increasing Fluo-3 fluorescence in the presence of ZnCl_2_ in C6 glioma cells, which occurred independent of the presence of added CaCl_2_. In addition, co-existence of pyrithione would render the fluorescence of Fluo-3, rather than FluoZin-3, unstable after the addition of A23187. FluoZin-3 (K_d_ = 15.0 nM) is shown to have much higher affinity and selectivity for Zn^2+^ than other indicators such as FluoZin-1 (K_d_ = 7.8 μM) and FluoZin-2 (K_d_ = 2.1 μM). This is one of the reasons why we used FluoZin-3 as a fluorescent dye to selectively detect intracellular free Zn^2+^ amongst different indicators in this study.

The current findings that exposure to ZnCl_2_ led to a similarly drastic inhibition of MTT reducing activity irrespective of the presence of added CaCl_2_ altogether give rise to an unexpected idea that all Ca^2+^-sensitive reagents used here would substantially interact with Zn^2+^ besides Ca^2+^ in cultured C6 glioma cells *in vitro*. If EGTA were indeed a chelator selective for Ca^2+^ rather than Zn^2+^, the inhibition by ZnCl_2_ should not have been prevented by EGTA in the absence of added CaCl_2_. From a viewpoint of the prevention by EGTA of the cytotoxicity of ZnCl_2_ in the absence of added CaCl_2_, along with the failure of removal of CaCl_2_ to modulate the cytotoxicity, the possible potential interaction of EGTA with Zn^2+^ is highly conceivable. Similar unexpected potential interactions with Zn^2+^ were seen for Fluo-3, Rhod-2, A23187 and BAPTA, all of which have been widely used to evaluate and confirm the involvement of free Ca^2+^ in physiological, pharmacological, cellular and molecular mechanisms underlying a variety of cell biological phenomena for several decades. Taken together, much attention should be carefully paid to *in vitro* pharmacological profiling using these Ca^2+^-sensitive reagents for validation of a role of free Ca^2+^ in physiological and pathological processes in diverse tissues enriched of endogenous Zn^2+^.

In contrast to a variety of Ca^2+^-sensitive reagents used here, the present findings give support for an advantage of the usage of several chemicals as a reagent selective for Zn^2+^ rather than Ca^2+^. The absolute requirement for ZnCl_2_ by pyrithione to rapidly increase both Fluo-3 and Rhod-2 fluorescence even in the presence of CaCl_2_ argues in favor of an idea that pyrithione is an ionophore highly permeable for Zn^2+^ with an ability to increase the fluorescence intensity of both Fluo-3 and Rhod-2. The failure of TPEN to inhibit the elevated fluorescence intensity of both Fluo-3 and Rhod-2 in acquired NMDAR channels in the presence of both agonists is suggestive of higher selectivity of this chelator for free Zn^2+^ than Ca^2+^. Similar intensification of FluoZin-3 fluorescence irrespective of added CaCl_2_ in an EGTA-sensitive manner gives support for the usefulness of this fluorescent indicator for selective determination of intracellular free Zn^2+^ concentrations. Extracellular and/or intracellular Zn^2+^ could be at least in part responsible for the molecular pathogenesis of different neurodegenerative and neuropsychiatric disorders besides Ca^2+^ in a particular situation. Zinc is condensed in synaptic vesicles along with Glu for subsequent exocytotic release into synaptic clefts upon stimuli in glutamatergic nerve terminals in the brain [39–41]. Zinc metabolism is shown to be at least in part responsible for the pathogenesis of Alzheimer’s disease [25,42], whereas Zn^2+^ plays a role in mechanisms underlying selective neuronal cell death after transient global cerebral ischemia in rats [43].

The current findings on possible inadequacy of a variety of chemicals well known for years as tools for the specific chelation and/or detection of free Ca^2+^ in different cells undoubtedly discourage the use of these familiar chelators, ionophore and fluorescent dyes for free Ca^2+^ in different biological materials. The intracellular free Zn^2+^ concentration is reported to be below nM ranges in different cells including neurons as seen with intracellular free Ca^2+^, moreover, while intracellular free Zn^2+^ is shown to be accumulated into a variety of organelles including endoplasmic reticulum by particular zinc transporters [38–40]. The possibility that intracellular free Zn^2+^ plays a role in the physiology and pathophysiology in a manner similar to intracellular free Ca^2+^ in the brain is not ruled out. The use of a chelator, ionophore and fluorescent dye selective for free Zn^2+^ could be beneficial for the future discovery and disclosure of novel insights into elucidation of the role of intracellular free divalent cations in a variety of brain functions. Taking into consideration clearly distinct emission spectrum profiles of the three different dyes bound to the corresponding divalent cations, however, possible fluorescence interference is unlikely within the fluorescent dyes used.

It should be emphasized that all neural cell lines employed here exhibited high vulnerability to the toxicity of ZnCl_2_ in an EGTA-sensitive fashion together with *mRNA* expression of several Zn^2+^ transporters. In particular, vulnerable cells invariably expressed *Slc39a* family members such as *Slc39a6* and *Slc39a7* rather than *Slc30a* family members. Intracellular Zn^2+^ levels are sophisticatedly regulated by both influx and efflux mediated by two types of membrane transporters for this divalent cation across cell membranes, in addition to metallothioneins [44]. The SLC39 family is believed to play a role in cellular Zn^2+^ homeostasis as Zn^2+^ exporters across cell membranes, while the view that the SLC30 family is responsible for the influx of Zn^2+^ as an importer is prevailing [45–47]. SLC39A6 is shown to mediate the incorporation of extracellular Zn^2+^ in SH-SY5Y neuroblastoma cells [48], however, whereas SLC39A7 regulates mobilization of Zn^2+^ from Golgi apparatus to the cytoplasm [49]. The possibility that particular Zn^2+^ transporters are at least in part involved in the cellular vulnerability seen after brief exposure to ZnCl_2_ in different neural cells is thus not ruled out so far. The fact that removal of added CaCl_2_ failed to affect MTT reduction in C6 glioma cells exposed to ZnCl_2_ at all, by contrast, does not give rise to an involvement of the incorporation of extracellular Ca^2+^ across cell membranes in the cytotoxicity mediated by Zn^2+^.

It thus appears that the use of Ca^2+^-sensitive reagents widely used for years is insufficient for the direct demonstration of participation of this divalent cation in a variety of molecular, cellular and biochemical events in a particular situation. A comprehensive analysis on both Ca^2+^ and Zn^2+^ could be at least required for the accurate validation and identification of bioactive divalent cation responsible for diverse biological activities in different plasma cells enriched of Zn^2+^.
